# Normative convergence between global health security and universal health coverage: a qualitative analysis of international health negotiations in the wake of COVID-19

**DOI:** 10.1186/s12992-025-01099-3

**Published:** 2025-02-24

**Authors:** Arush Lal, Clare Wenham, Justin Parkhurst

**Affiliations:** Department of Health Policy, London School of Economics & Political Science, London, UK

**Keywords:** Global health diplomacy, Global health security, Universal health coverage, Politics, Governance, Health systems, Negotiations, International affairs, Norms

## Abstract

**Background:**

The UN Sustainable Development Goals (SDGs) and WHO Thirteenth General Programme of Work underscored the importance of mitigating health emergencies while ensuring accessible and affordable health services. Central to these efforts are global health security (GHS) and universal health coverage (UHC), which act both as standalone goals and as cross-cutting approaches to health policy and practice. While GHS and UHC each operate as distinct norms, global health stakeholders increasingly advocate for advancing them synergistically to address interconnected health challenges amid limited resources. However, the current extent of alignment between GHS and UHC remains unclear, especially post-COVID-19. This qualitative study assesses normative convergence between GHS and UHC by tracing their development through iterative draft texts across two major international health negotiations – specifically examining how UHC norms are expressed in the WHO Pandemic Agreement, and how GHS norms are expressed in the 2023 UNGA Political Declaration on Universal Health Coverage.

**Results:**

UHC was promoted in the WHO Pandemic Agreement through three closely-associated discourse themes (rights-based narratives, equity frames, focus on social determinants of health) and three closely-associated core functions (accessible and affordable health commodities, prioritizing vulnerable populations, primary health care approach). Meanwhile, GHS was reciprocally promoted in the 2023 UHC Political Declaration through three related discourse themes (existential threat narratives, resilience frames, focus on infectious diseases) and three related core functions (outbreak preparedness, health emergency response, One Health approach).

**Conclusions:**

The findings indicate that the COVID-19 pandemic created a policy window uniquely-positioned to accelerate normative convergence between GHS and UHC. Both international agreements advanced convergence by demonstrating increased complementarity and interdependency between the two norms through the co-promotion of their underlying features. However, negotiators agreed to political and operational trade-offs which made it difficult to sustain progress. This study provides a nuanced account of how global health norms evolve through integration in complex policy environments – finding that normative convergence may not always be explicit, but rather implicit through incremental linkages in their underlying discourse and core functions. This research contributes to pragmatic efforts by global health actors seeking consensus amidst an era of polycrisis, and highlights the importance of navigating geopolitics and overcoming path dependencies. It also deepens scholarly understanding on how ‘hybrid norms’ develop through the dynamic process of normative convergence via diplomacy.

## Introduction

As public health has become a priority for international cooperation, researchers have increasingly sought to analyse the role of norms in shaping global health politics and practice. Norms are seen to capture broad ideas identified by collective understandings, organizing principles, and expected behaviors [1]. Given the influence of norms on both the conceptualisation of major health challenges and the policy solutions to address them, global health stakeholders often turn to international agreements to institutionalize and legitimize emerging health norms [1–3]. 

Previous studies have identified global health security (GHS) and universal health coverage (UHC) as two major concepts driving health policy [4, 5]. A subset of this literature has examined their associated norms separately, perceiving them to entail distinct sets of underlying discourse and core functions. For example, GHS typically emphasizes securitization frames to address infectious disease outbreaks [6], while UHC emphasizes rights-based frames through accessible and affordable health services [7].

Global health stakeholders have increasingly sought to identify synergies between GHS and UHC as a way to maximize limited resources while addressing multifaceted health challenges [8]. An emerging body of work has attempted to advance scholarship on the integration of GHS and UHC, viewing them not as separate, ‘fixed’ norms, but instead as dynamic ‘processes’ that are continually-evolving and contested [9]. By tracing how GHS and UHC have been (re)constructed over several decades, this view conceptualizes both as individual norms as well as broader normative regimes comprised of overlapping actors, policies, and governance structures that are more interconnected than previously thought. However, what has not yet been explored is how GHS and UHC norms converge, nor what their current state of normative alignment looks like in the wake of the COVID-19 pandemic. This has important implications for policy formulation in response to complex crises, as well as for fostering consensus through overlapping global health diplomacy mechanisms.

This paper addresses this gap by analysing the convergence between GHS and UHC norms to uncover recent shifts in their evolution and subsequent impact on global health policy. Through a multimethod qualitative analysis of two major international health agreements launched after the pandemic, we examine the positioning of specific GHS and UHC norms across successive negotiation drafts, thus helping determine how, to what degree, and to what level of sustainability there has been normative convergence. This mapping exercise, which focuses on an under-examined area of global health, holds important implications for health diplomacy and governance, where policymakers often grapple with how to reconcile fragmented policies and investments stemming from longstanding silo-isation [10] of public health initiatives. By providing an interpretation of how convergence processes unfold and to what end, our analysis helps to better understand how GHS and UHC, with their established histories of co-evolving reconstructions, can be pursued alongside each other as mutually-reinforcing norms. Furthermore, this work contributes to broader global health policy and international relations scholarship on the unique ‘politics of integration’ that may occur when two or more powerful norms are pursued, not as hierarchical or inherently incompatible, but rather through a dynamic (and ultimately more productive) process of strategic convergence as ‘hybrid norms’ via diplomacy.

## Background

### Brief review of GHS and UHC norms

Global health security (GHS) is defined as the activities required to minimize the impact of acute public health events across international borders [11]. GHS norms stem from historical linkages between health and security concerns [12], and tend to focus on risks to state interests necessitating international cooperation [13–15]. They often employ discourse themes such as existential threats [6], resilience [16], and infectious diseases [4]. Consequently, GHS norms are typically enshrined in agreements such as the International Health Regulations (2005) (IHR) [12], and operationalized through core functions related to outbreak preparedness [17], health emergency response [18], and a One Health approach [19].

Meanwhile, universal health coverage (UHC) is defined as ensuring all people have access to a comprehensive range of quality health services without posing financial hardship [20]. UHC norms stem from the recognition of a right to health [21], and tend to focus on gaps in local or community healthcare necessitating domestic health system strengthening [22]. They often employ discourse themes such as human rights [23], equity [24], and social determinants of health (SDH) [25]. Consequently, UHC norms are typically operationalized through agreements such as the UN Sustainable Development Goals (SDGs) [26], and include core functions related to prioritizing support for vulnerable populations [25], accessible and affordable health commodities [27], and a primary health care (PHC) approach [28].

Traditionally, global health actors have treated GHS and UHC as distinct concepts, highlighting fundamental differences in the principles and approaches underpinning them [29]. However, recent scholarship [30] argues that fragmentation between these concepts – often cyclically exacerbated by competition for attention [31] and vertically-siloed investments [32] – potentially leads to poorer health outcomes. This may be the result of divergent conceptualisations of ‘risk’ between GHS and UHC, leading to varying views on policy solutions [29]. Furthermore, priorities of ‘what’ to improve in health systems often differ between the two, thereby perpetuating divergence. Misalignment between GHS and UHC norms can be particularly detrimental during health emergencies, as witnessed in the 2014 West Africa Ebola outbreak [33] and the 2016 Zika epidemic [34], where disjointed and poorly coordinated health system interventions weakened response efforts. In the wake of these crises, and amidst broader resource constraints, global health actors have increasingly sought to align GHS and UHC, with WHO Director-General Ghebreyesus even characterizing GHS and UHC as “two sides of the same coin.” [35] Recent frameworks [36] and technical reports [37] published by WHO further demonstrate their efforts to operationalize coherent health systems that better connect GHS and UHC.

However, there persists a lack of clarity on how to effectively harmonize GHS and UHC norms, thus posing significant challenges to public health implementation. Researchers point out that, “although WHO approaches [GHS and UHC] agendas in principle as imminently convergent inputs towards a strong health system, scarce resources and political realities force policymakers to make tough choices,” leading to prioritisation of one over the other [30]. Therefore, understanding the current extent of convergence between GHS and UHC norms – particularly in the wake of a crisis – provides important implications for the way both are pursued moving forward, with repercussions for which policies are prioritized by whom, at what levels of investment, and with which types of governance arrangements.

### Current context

The context of international negotiations provides crucial insights into the challenges facing states, motivations for crafting specific commitments, and the normative landscape surrounding diplomatic efforts. Because this study focuses on the state of convergence between GHS and UHC norms following the COVID-19 pandemic, it is crucial to first appreciate recent trends in their development prior to the crisis and in its immediate aftermath.

The global health landscape witnessed significant normative shifts in response to the SDGs and post-2015 development agenda. The midpoint of the MDGs (circa 2007–2010) was marked by various stocktaking initiatives and strategic realignments following perceived failures of the MDGs [38]. Changes to global health initiatives during this period had notable implications on GHS and UHC norms, such as the push from vertical disease programming to horizontal health system strengthening [32] and an emphasis on financial protection for health services [20]. This transition laid the groundwork for (re)constructed GHS and UHC norms that were eventually reflected in the SDGs, alongside the promotion of normative frames such as ‘sustainability’ that have since heavily influenced contemporary global health policy [39]. Echoing this introspective phase observed halfway through the MDGs, global health stakeholders similarly found themselves grappling with still-unresolved ‘wicked problems’ as they approached an analogous midpoint – the 2030 SDGs deadline – just as the first cases of COVID-19 were reported.

The COVID-19 pandemic, which began in early 2020, revealed new dynamics in GHS and UHC which reflected vulnerabilities in both frameworks [37]. In the realm of GHS, the pandemic ushered outbreak responses through heavily securitized discourse [40]. The crisis exposed gaps in conventional GHS core capacities (e.g., surveillance, zoonotic spillover, early warning and alert), but also significant weaknesses in health systems that undermined existing GHS functions (e.g., disruptions to essential health services, inequitable delivery of pandemic countermeasures, poor community engagement) [41]. Conversely, within the UHC domain, the pandemic catalyzed attention on equity and access through rights-based discourses [42]. Chronic gaps in affordable healthcare and SDH were brought to the forefront, while exposing neglected shortcomings (e.g., inadequate health worker protections, disjointed emergency management, and complications due to noncommunicable diseases) [43, 44]. The pandemic therefore demonstrated how pervasive fragmentation across health systems necessitated urgent and comprehensive reforms to global health governance, including between GHS and UHC.

In response to the challenges posed by COVID-19, various efforts were launched to mitigate the ongoing crisis and address future public health challenges. The Working Group on Strengthening WHO Preparedness and Response to Health Emergencies played a pivotal role in synthesizing emerging lessons by recommending amendments to the IHR (2005) and establishing an International Negotiating Body (INB) to develop a new instrument on pandemic prevention, preparedness, response, and recovery (the ‘Pandemic Agreement’) [45]. Member States intentionally designated the scope of the INB to extend beyond the purview of existing IHR in order to address GHS gaps through a novel mechanism capable of strengthening equity and global solidarity in future pandemics.

Simultaneously, the 2023 UN General Assembly (UNGA) High-Level Meeting on Universal Health Coverage, long-planned as a follow-up to the landmark 2019 UNGA High-Level Meeting (HLM) on Universal Health Coverage, gained renewed significance in the aftermath of COVID-19 [46]. Its resulting Political Declaration grappled with challenges exposed by the crisis and stalled progress to achieve UHC targets by 2030. Notably, numerous other efforts were also underway which further complicated the normative landscape, including simultaneous planning for two other health-related HLMs during the same UNGA – one on Pandemic Prevention, Preparedness, and Response (PPR) and the other on Tuberculosis.

## Conceptual Approach and methods

The WHO Pandemic Agreement (PA), and the 2023 UNGA Political Declaration on Universal Health Coverage (PD) present two key international negotiations in the wake of COVID-19 through which to analyse the ways in which GHS and UHC norms have been shaped – and are ultimately converging.

### Conceptualising norms

In international relations, norms are seen to encompass a spectrum of shared values and standardized procedures that shape interactions among State and non-State actors [1, 47]. Analyzing the evolution of norms can therefore offer fresh perspectives on various intersecting, complementary, and oppositional understandings of what *is* happening versus what *ought* to happen in complex policy environments like global health [3]. International actors contend that in order for norms to be strengthened or “seen as legitimate,” they must first gain widespread acceptance, often through legal codification [2]. This makes international agreements or treaties particularly useful for tracing normative shifts.

A discursive approach [48] focused on norms as “sensemaking practices” [49] offers helpful insights to examine patterns in their origins, adoption, and operationalization. As Epstein argues, discourses shape what people do and who they are by fixing meanings and opening subjective spaces through which norms are developed [50]. A discursive approach helps highlight the active role that global health actors play in reinscribing particular normative concepts (e.g., relevant frames or policies) through legal mechanisms via global health diplomacy [51]. This study therefore conceptualizes GHS and UHC norms as evolving ‘processes’ [49], adopting this discursive approach to better appreciate the relative weightings of various discourses as well as subtle changes in the deployment of these discourses across both international agreements.

Norms are traditionally analysed through expressions of core ideals and value statements. However, in contexts where the exercise of norms is inextricably linked with technical interventions, it may also be necessary to examine how they are operationalized through specific legal or policy commitments [26]. For example, Drope and Lencucha argue that the operationalization of norms fundamentally shapes discourse, thus further influencing norm development [3]. Therefore, this analysis conceptualizes norms as comprised of normative frames and guiding principles (referred to as ‘discourse’) as well as resultant capacities and policy actions central to implementation (referred to as ‘core functions’). This approach recognizes that as GHS and UHC evolve to take on new meanings, so too do the activities they are seen to encompass. In doing so, we aim to provide a fuller account of GHS and UHC norm development, including how ideas and frames define the set of health services expected, and how prioritization of specific health issues leads to new obligations for stakeholders.

### Defining norm convergence

Few scholars have examined in-depth the specific form of norm convergence analysed in the context of this study. For example, much of the available literature on norm convergence focuses on the diffusion of a particular norm across multiple institutions [52], or the integration of multiple policies across a broader norm regime or context [53]. However, limited research examines the convergence of two relatively distinct (yet equally influential) norms and their associated regimes, nor clearly traces this form of normative convergence through diplomatic forums. Drawing on existing conceptualizations of norm convergence, we provide a new definition that is more appropriate for this type of analysis.

Convergence, as described by Knill, refers to the tendency of policies to “develop similarities in structures, processes, and performances.” [54] Scholars further contend that convergence usually entails “moving from different positions toward some common point,” [55] as well as what Mende refers to as “complementarity […] via inclusion” of previously-external elements [56]. Furthermore, international agreements are often considered as useful contexts for examining convergence, given that nations utilize these mechanisms to jointly address cross-border issues [52, 55]. Therefore, one aspect of normative convergence may be indicated by similarity over time, evidenced through increased complementarity following inclusion of previously-distinct norms within international agreements.

Another element of norm convergence involves integration through a “shared normative framework.” [56] Candel and Biesbroek describe integration as a process in which “constituent elements are brought together and made subject to a single, unifying conception.” [57] Tosun and Lang extend this to suggest that “certain domains take policy goals of other, arguably adjacent, domains into account,” [58] thereby creating “interdependencies between different policy sectors and [then coordinating] these.” Thus, another aspect of normative convergence may be indicated by interdependency, evidenced through increased interlinkages with an awareness of cross-sectoral implications.

Norm convergence can therefore be defined as a process in which there is demonstrated commitment to a shared normative framework, through meaningful incorporation of distinct norms across reciprocal domains as well as integration of underlying discourse and core functions. In the context of this study, norm convergence can be evidenced within the international agreements by clear: (1) complementarity (e.g., diffusion of UHC norms within the PA and diffusion of GHS norms within the PD), and (2) interdependency (e.g., interlinkages between GHS and UHC norms that demonstrate cross-sectoral awareness).

### Methodological approach

#### Case selection

The specific cases of the PA and PD were selected based on two criteria. First, the agreements were negotiated through the WHO and UNGA, respectively – two institutions recognized as the most prominent mechanisms for global health diplomacy [3]. Both intergovernmental organizations are alike in their liberal democratic view of international health cooperation, but also diverge slightly between core mandates, governance procedures, and internal politics–providing complementary locations to examine normative development. Second, both agreements were perceived by global health actors as significant forums to address health systems gaps made prominent by the COVID-19 pandemic – with the PA serving as one of the most high-profile efforts to codify reforms to GHS, and the PD serving as one of the most high-profile efforts to redress inaction on UHC [59]. Notably, the adoption of both agreements necessitated achieving consensus through similar processes following multiple draft revisions, leading to discursively-rich debates on normative positions.

Taken together, the two case studies offer a unique opportunity for nuanced analysis of how norms may shift through integration. We thus use a within-case comparative design [60], enabling insights from cross-case variation, while retaining comparability between cases due to similar background conditions. For example, the WHO-led INB process was more deeply embedded in the GHS regime, having been specifically initiated to address inadequate pandemic preparedness and response mechanisms as well as related capacities outside the scope of the IHR (2005) [61]. Meanwhile, the UN-led UHC-HLM negotiations were heavily rooted in UHC norms, given the aim of advancing right-to-health obligations and re-invigorating stalled progress since the 2019 UHC-HLM [62]. The intentional insertion of UHC norms into the PA texts and GHS norms into the PD texts would therefore suggest noteworthy changes in the way stakeholders conceptualize the scope of both, thereby demonstrating normative evolution and convergence. Neither mechanism has been studied with regard to its potential impact on the convergence of GHS and UHC; assessing both together provides novel insights on how integration may occur between these influential agendas.

#### Data collection and analysis

Adapting methodological approaches outlined by Alejandro and Zhao, we applied a two-step process comprising of a qualitative content analysis (QCA) – which includes “systematically classifying material by assessing the presence/absence and frequency of relevant elements” – alongside discourse analysis – which helps “unpack the linguistic mechanisms at play and their potential socio-political effects.” [63] By conducting QCA (to provide breadth) in parallel with discourse analysis as the analytical framework (to provide depth), we were able to reveal both “the components of interest,” as well as “inconsistencies or implicit meanings with regards to attitudes.” [64] As Alejandro and Zhao note, “for QCA, the addition of discourse analysis can bring a critical perspective to investigate meaning in context, while for discourse analysis, the addition of QCA can provide a broad dataset to help researchers focus on the temporal and spatial changes in discourse.” [63] This is consistent with previous studies that examine shifting policies and normative positions, which similarly set out to understand “the extent to which various discourses [are] deployed across the data set and changes in usage over time.” [65].

The first step of the analysis involved the QCA, which examined content from official documents published in WHO and UN repositories related to the INB and UHC-HLM. Specifically, the primary data for analysis centered on the first six successive negotiation drafts pertaining to the development of the PA, and five successive negotiation texts culminating in the adopted PD. Guided by a similar method demonstrated by Hsieh and Shannon [66], and developed as part of a wider study [67] on the integration of GHS and UHC norms, the directed QCA began with a scanning of all primary documents and related literature on GHS and UHC to identify key terms as guidance for initial codes (summarised in Table 1). Representing dominant expressions of GHS and UHC norms, these terms were organized into discourse and core functions by considering the “categorization, subject positions, rhetorical strategies, and lexical fields” enabled by a discursive reading of the documents [64].

**Table 1:**
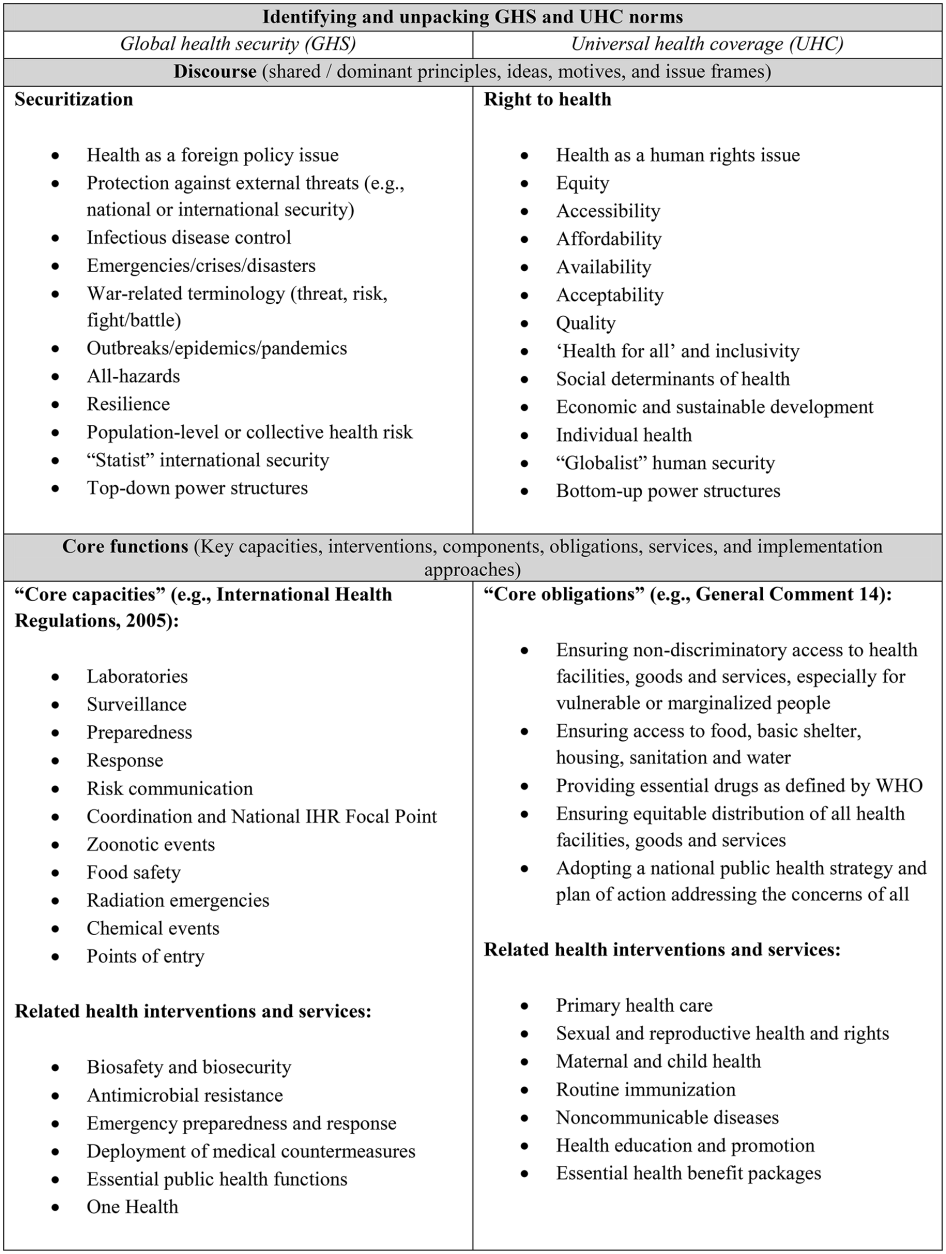
Key terms related to the norms of global health security and universal health coverage, including dominant discourse and commonly-associated core functions of each (non-exhaustive list)

Having completed this initial list of specific forms of GHS and UHC discourse and core functions to search for, we could then proceed by identifying emerging patterns of normative positions across both sets of negotiation texts, classifying relevant text segments into thematic categories (see Table 2). This deductive search aimed to qualitatively identify the presence and frequency of discourse and core functions from one domain across reciprocal draft texts of the other (i.e., UHC norms within PA drafts and GHS norms within PD drafts). Through this directed QCA, we were able to determine major themes in the dominant expressions of GHS and UHC over successive drafts.

**Table 2:**
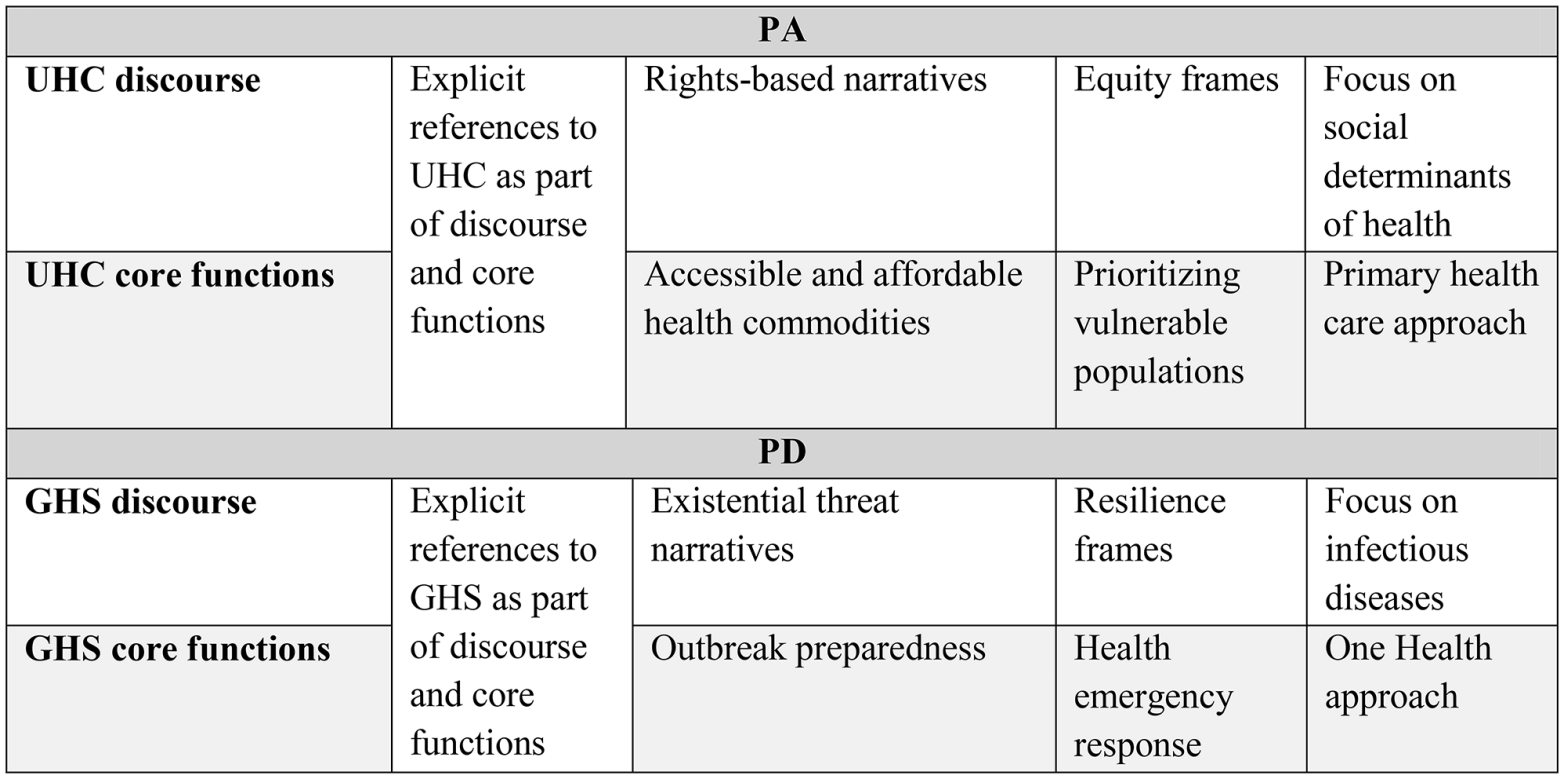
Emerging themes covering key expressions of universal health coverage found in the Pandemic Agreement and global health security found in the Political Declaration

The subsequent step involved utilizing discourse analysis to more critically unpack the normative themes identified from negotiation texts. In presenting our findings, we discursively analyzed relevant text segments that expressed the thematic categories, with an emphasis on revealing “the shifts, changes, and the pervasiveness of particular positions” [65] across successive drafts. In line with our definition of norm convergence, this involved identifying examples of similarity over time (complementarity) and cross-sectoral awareness (interdependency). Together, evidence along both dimensions would demonstrate advancement of a shared normative framework, thus indicating normative convergence.

Because normative themes examined across iterative draft revisions were often subject to significant “variability, contradiction, and tension,” [65] the same terms were occasionally used in different ways and at different times. Thus, the focus of this dual-faceted analysis was to qualitatively explore how often normative positions were included and repeated across drafts (presence, frequency), but more importantly, how they were expressed in context (weight, centrality, implicit dimensions). This approach enabled us to systematically explore the depth *and* breadth of GHS and UHC norms as they were either mainstreamed, transformed, or altogether excluded across iterative negotiation drafts – from initial concept to final available text. In this way, we drew novel insights on the evolution and extent of normative convergence between both norms across these significant international agreements.

## Results

### UHC norms in the Pandemic Agreement

Six iterative negotiation drafts pertaining to the development of the Pandemic Agreement were analysed (Table 3):

**Table 3:**
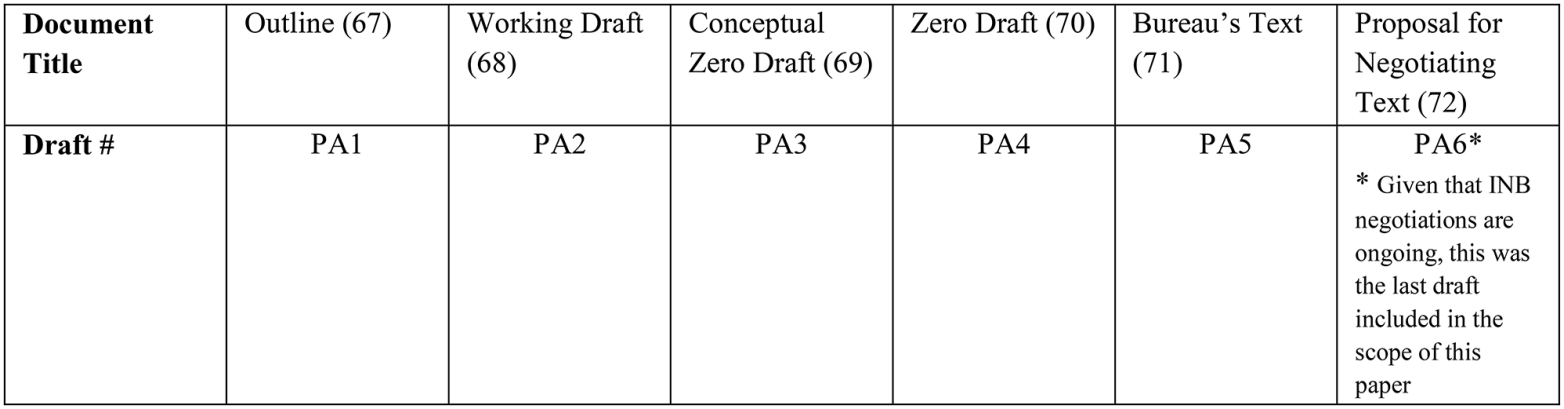
Document title and corresponding draft abbreviation used for analysis of UHC norms in the Pandemic Agreement

## Explicit references to UHC

One of the clearest ways to identify normative convergence with UHC in the GHS-focused Pandemic Agreement is through explicit references to ‘universal health coverage.’ Overall, direct references to UHC generally increased in prominence until the Zero Draft (PA4), after which they somewhat diminished.

While all negotiation texts appear to link UHC with GHS, these references become less explicit in later drafts. PA1 calls for “resilient health systems for UHC and health security,” while the PA4 shifts language to “*recognize* the need for resilient health systems, *rooted in* UHC” to mitigate pandemic shocks (not “health security”); PA6 ultimately calls for each Party to “strengthen its health system” for sustainable PPR, “*with a view to the progressive realization* of UHC” [emphasis added]. Relatedly, PA2 reiterated “universal health coverage as an essential foundation for effective pandemic prevention, preparedness and response” – a phrase repeated in the subsequent drafts. Although PA6 elevated this point higher in the preamble (suggesting increased importance), it was no longer framed as a “foundation” for PPR.

Early drafts signaled that the PA would be “guided by the goal of achieving UHC as an overarching principle.” [69] This was iteratively amended to “the *aim* of achieving UHC,” [71] [emphasis added] until PA6 excluded UHC as a guiding principle (though it remained defined as a key term). A similar pattern played out in revised objectives statements, with initial drafts committing to “a view to achieving UHC,” [69] followed by “the progressive realization of UHC,” [72] and the ultimate removal of “universal health coverage” from the scope of work.

Initial drafts warned that the “disproportionally heavy impact” [69] of pandemics “hamper[ed] the achievement of universal health coverage” and emphasized related UHC ideas like “equitable access to high quality health services without financial hardship.” [70] These were largely cut by PA6. This fluctuation corresponded to changes in the types of interventions linked with UHC, with PA1 advocating for “access to quality, agile, and sustainable health services for universal health coverage,” PA2 expanding to include clinical and mental care, PA3 calling for “continuity of PHC and UHC” by “maintaining” service availability and addressing backlogs – yet later texts reduced these explicit mentions of UHC capacities.

Finally, almost all drafts discuss some version of “enhanced collaboration between the health and finance sectors in support of UHC, and as a means to support [PPR].” [68] One interim text urges the enhancement of financial and technological assistance “to strengthen health systems consistent with the goal of [UHC],” [72] which is largely retained by PA6 but caveated by “within available means and resources.” Meanwhile, a PA4 commitment to “prioritize and increase or maintain” domestic funding on PPR emphasizes “working to achieve UHC,” while PA6 ultimately excludes such direct references to UHC.

### UHC discourse

Overall, there were three main ways that UHC discourses were expressed across draft texts of the PA: (1) rights-based narratives, (2) equity frames and (3) a focus on SDH.

## Rights-based narratives

Human rights narratives are prominently featured across PA drafts. For example, all texts from PA2 through PA6 evoke the WHO Constitution, stating that “the highest attainable standard of health is one of the fundamental rights of every human.” However, a distinction is drawn over successive drafts between “respect for human rights” (appearing in all versions) and the “right to health” (appearing until PA4 as a guiding principle, yet removed in PA5). This shift in language appears to alleviate concerns around obligations to “protect and promote” the right to health, which is also absent by PA6.

Expressions of other rights-based narratives further demonstrate principles commonly associated with UHC. For example, “inclusiveness” is defined in all texts after PA2 as “the full and active engagement with, and participation of, communities and relevant stakeholders across all levels.” Other related examples include references to community engagement, gender equality, nondiscrimination, and respect for diversity. Though PA5 neglects to individually name these principles, it instead retains a broader section on “people in vulnerable situations,” under which these concepts are implicitly grouped.

## Equity frames

Equity frames are largely promoted in two discursive ways. First, equity is explicitly framed as a “cross-cutting strategic theme,” [68] with interim drafts arguing that “equity should be a principle, an indicator and an outcome of pandemic prevention, preparedness and response.” [71] Equity is characterized in PA6 as the “centre of [PPR],” reflected in calls for “unhindered, fair, […] access to […] affordable pandemic-related products and services […] and social protection” – providing linkages to conventional UHC discourses. Second, equity is promoted as an underlying principle for the operationalization of the PA, serving as a departure point for broader concepts seen to improve solidarity during pandemics. For example, the principle of “common but differentiated responsibilities” (CBDR) is repeated throughout draft texts, urging states to implement PPR measures that consider “the specific needs and special circumstances of developing country Parties” and that “Parties that hold more capacities and resources relevant to pandemics should bear a commensurate degree of differentiated responsibility” [71] (n.b., while PA5 softens CBDR provisions to instead “provide such support voluntarily,” they remain rooted in equity).

## Social determinants of health

Draft texts across the PA underscore UHC discourse themes related to SDH, offering broader links to health promotion and intersectoral collaboration. PA1 emphasizes the objective to “save lives and protect livelihoods,” a sentiment preserved throughout successive drafts. Acknowledging the “catastrophic health, social, economic and political consequences” of pandemics, PA2 urges “action on social determinants of health […] by a comprehensive intersectoral approach” and a “whole-of-society” perspective that considers PPR impacts on “economic growth, employment, trade, transport, gender inequality, food insecurity, education and culture.” PA4 even alluded to SDH in its definition of “pandemic,” noting “social and economic disruptions” and emphasizing “resolute action on social, environmental, cultural, political and economic determinants of health.”

Later drafts advance UHC discourse via SDH through commitments to One Health, such as recognizing the “interconnection between people, animals, plants and their shared environment” and acknowledging “that economic and social development and poverty eradication are the first and overriding priorities of developing country Parties.” PA6 further mainstreams SDH, advocating for “clean water, energy and air, safe and nutritious food, taking action on climate change, and contributing to sustainable development” in the PA.

### UHC core functions

Core functions of UHC provide particular insights into how UHC is being operationalized as specific actions. These can be grouped in three ways: (1) accessible and affordable health commodities, (2) prioritizing vulnerable populations, and (3) a PHC approach.

## Accessible and affordable health commodities

One of the primary ways UHC is operationalized in the PA is through commitments to ensure “timely access to affordable, safe and effective pandemic response products.” [69] This is echoed by interim drafts, which call for a “coordinated approach to the availability and distribution of, and equitable access to, pandemic response products” [70] as well as the development of a mechanism to ensure their “fair and equitable allocation.” [71] PA6 proposes giving WHO “real-time access” to 20% of production of these products, and advocates for cost-related arrangements such as “tiered-pricing” based on country income levels.

Efforts to ensure affordable access healthcare commodities extend to “health technologies that promote the strengthening of national health systems and mitigate social inequalities.” For example, later drafts propose a WHO Pathogen Access and Benefit-Sharing System (PABS) System – a mechanism to promote the rapid and transparent sharing of pandemic pathogens and genetic data while ensuring fair access to the resulting benefits [70]. Related capacities commonly associated with UHC also include “time-bound waivers of intellectual property rights,” [72] technology transfer, “training of clinical research networks” [73], regulatory approvals for quality and safety, and cost and pricing transparency.

## Prioritizing vulnerable populations

All PA drafts demonstrate varying commitments to prioritize vulnerable populations – an obligation inherent in previous texts foundational to UHC, such as General Comment 14 [74]. The PA1 emphasizes resource allocation “based on public health need” and a “policy to safeguard vulnerable populations most affected by pandemics.” Subsequent drafts expand this to include “access to pandemic response products by […] frontline workers” [69] as well as refugees, the elderly, persons with disabilities, pregnant women, and infants [70]. PA5 ultimately streamlines these references upfront under “persons in vulnerable situations,” characterizing neglect of their needs as “threats and barriers to the full realization of the right to health.”

Capacities linked to this UHC theme are seen in references to “equitable gender, geographical and socioeconomic status representation and participation.” [68] Another draft advocates for inclusive policies for women health workers and “addressing discrimination, stigma and inequality” with “data disaggregated by gender.” [70] PA4 emphasizes “gender equality” as a guiding principle and calls to center “youth and women,” while PA5 calls for further data disaggregation by “age, geography, socioeconomic status.” PA6 stresses that clinical trials consider “racial, ethnic and gender diversity across the life cycle.”

Community engagement, another function historically linked to UHC, receives mixed uptake. Building on an earlier draft urging “measures to mobilize social capital in the community […] especially to vulnerable populations,” [69] PA3 underscores community engagement to ensure “ownership of, and contribution to, community readiness and resilience.” PA4 further calls for national multisectoral mechanisms “with meaningful” community representation. However, PA5 introduces caveats such as “in keeping with national capacities” and “as appropriate” when discussing engagement with civil society. Ultimately, PA6 only explicitly references community engagement in articles on R&D, One Health, and whole-of-society approaches.

## Primary health care approach

Another way UHC is expressed in the PA is through commitments to a PHC approach. PA1 emphasizes “access to lifesaving, scalable and safe clinical care […and…] continuity of health services and palliative care.” A subsequent draft urges financing to “maintain and restore routine public health functions” and “prevention strategies for epidemic-prone diseases.” [70] PA4 reiterates “a focus on [PHC] and community-level interventions,” echoed in PA5 that calls for “rehabilitation and post-pandemic health system recovery.” However, PA6 removes some PHC capacities while simultaneously enhancing a focus on “essential” health services.

Capacity-building for service delivery further advances UHC through a PHC approach, which PA2 states is “core to achieving and sustaining [PA] objective(s).” PA1 stresses “an adequate number of health workforce with public health competency” and “mobile laboratories [and] diagnostic networks.” Subsequent drafts expand these commitments, with PA6 calling for “coordinated data interoperability,” “integrated public health surveillance,” and prevention of “violence and threats against health workers.” Yet, PA6 omits previous language [70] on universal forecasting platforms, “engagement of communities in surveillance,” and safeguards against “substandard and falsified medical products.”

A third way that UHC is advanced through a PHC approach is by focusing on intersectoral collaboration in health systems, reflecting commitments enshrined in the 1978 Alma-Ata Declaration on PHC [75]. PA1 emphasizes “comprehensive multisectoral” PPR strategies, including for “infection prevention and control, water, sanitation and hygiene, antimicrobial resistance, transfer and treatment of patients, travel and movement of frontline workers” as well as multistakeholder engagement to include threats “resulting from climate change and environmental factors.” Subsequent iterations narrowed this language, such as only covering pathogens under the IHR in multisectoral public health surveillance or omitting “timely access […] for diagnosis or treatment.” [73] Despite this, PA6 continues to “promote and enhance synergies between multisectoral and transdisciplinary collaboration,” including by strengthening “science, public health and pandemic literacy [to] combat false, misleading, misinformation or disinformation.”

### GHS norms in the UHC-HLM Political Declaration

Five iterative documents relevant to the UHC-HLM Political Declaration negotiations were compiled (Table 4):

**Table 4:**
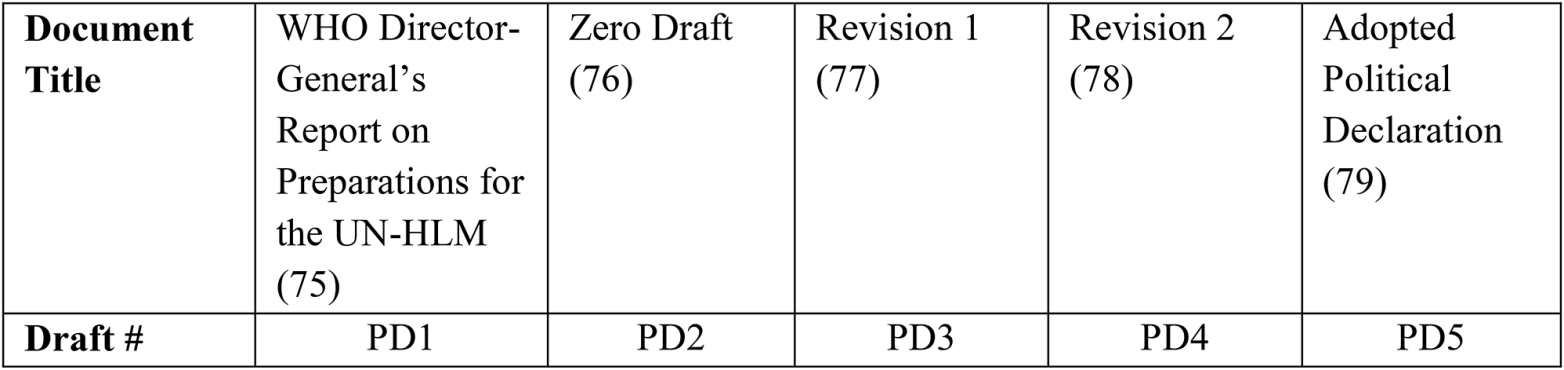
Document title and corresponding draft abbreviation used for analysis of GHS norms in the UHC Political Declaration

## Explicit references to GHS

Among the various drafts of the Political Declaration, only PD1 explicitly references ‘global health security’ as a discourse theme. It does so by prominently featuring the heading: “reorient unified national health systems towards primary health care as a foundation for universal health coverage, health security and better health.” Although subsequent iterations do not directly mention GHS as a guiding concept, its impact as a discourse strategy is still retained in other ways described below.

Meanwhile, although PD1 also stands out as the only draft to explicitly reference GHS capacities, it does so rather prominently. For example, it emphasizes that “scaling up and sustaining essential public health functions are vital to the recovery and resilience of national health systems for UHC and health security,” asserting that PHC “explicitly […] provides this integrative link.” Furthermore, PD1 identifies ongoing initiatives, programs, and actors contributing to “reorienting health systems to PHC as a foundation for UHC and health security.” These range from WHO programmes to other major development partners at global, regional and country levels reviewing “progress towards UHC and related issues concerning health security.” It also mentions the involvement of global and regional economic and financial institutions (e.g., World Bank, International Monetary Fund) that encourage “long-term, sustainable investment in UHC and health security.” While subsequent PD drafts do not directly cite GHS, there remain numerous linkages to core functions.

### GHS discourse

Overall, there were three main ways that GHS discourses were expressed across draft texts of the PD: (1) existential threat narratives, (2) resilience frames, and (3) a focus on infectious diseases.

## Existential threats

PD1 opened with a focus on existential threats to health and state security, noting a backdrop including the COVID-19 pandemic alongside “crises resulting from climate change and natural disasters, national and regional conflicts, profound economic recession” which impact “the health and well-being of the world’s 8 billion people.” It emphasized countering “inequalities among and within countries […] through global solidarity,” and “aligned collective action at the halfway point to the 2030 Agenda for Sustainable Development.” The subsequent PD2 urged “[strengthened] international cooperation” in response to “serious concern” over vaccine disparities hindering global COVID-19 control efforts. PD3 emphasized health financing bolstered by “national, regional and multilateral initiatives” to recover from pandemics, while PD4 underscored that “humanitarian emergencies and armed conflicts have a devastating impact on health systems” which expose vulnerable populations “to preventable diseases and other health risks.” Finally, PD5 further stressed “the global concern about the high prices of some health products,” recognizing that “inequitable access to such products impedes progress towards achieving UHC,” thus urging international cooperation particularly to mitigate the risk this poses to developing countries through securitized discourse.

## Resilience frames

Another emerging GHS discourse theme is the promotion of resilience frames [81]. The opening sections of PD1 emphasize that the UHC-HLM “presents an opportunity to go beyond the status quo” to “build resilience against global shocks,” thereby ensuring “preparedness for pandemics and other crises, including climate change.” PD1 further recognizes essential service delivery as “central to countries’ recovery from previous conflicts and crises,” a point echoed by the subsequent PD2, which notes an “increasing number of complex emergencies is hindering the achievement of UHC” and introduces risks like “the adverse impact of climate change, natural disasters, extreme weather events” to advocate for “resilient and people-centred health systems.” Its call for “a whole-of-government and health-in-all-policies approach,” is reflected in subsequent drafts, including PD3 which stresses “water, sanitation, hygiene and electricity services in health care facilities for health promotion, disease prevention” and PD4 which urges “a coherent approach to strengthen the global health architecture as well as health system resilience and UHC,” underlining linkages to PPR and One Health. Finally, all drafts affirm health workers as “as fundamental to strong and resilient health systems,” although PD5 tones down related language on climate change impact and community engagement.

## Infectious diseases

The PD also employs narratives on infectious diseases and their impacts, with PD1 cautioning that “countries continue to rely on fragmented disease and service-specific programmes and interventions.” It also notes that the “COVID-19 pandemic took a significant toll on progress towards the SDGs,” highlighting that the “combined macroeconomic, fiscal and health impact of COVID-19 point to worsening of financial protection globally.” By arguing that “experiences from COVID-19, Ebola virus, conflicts and disasters in 2022 have demonstrated that this requires multisectoral, whole-of-government action,” PD1 sets the stage for PD2, which cites mixed progress on major communicable diseases like HIV/AIDS, tuberculosis, malaria, and antimicrobial resistance as justification for PD negotiations. PD3 added language on the “importance of pandemic prevention, preparedness and response as a key component of UHC.” All subsequent drafts emphasized the “importance of coordination” and “promoting alignment and synergies across […] the High-level Meetings on Tuberculosis and Pandemic Prevention, Preparedness and Response” taking place alongside the UHC-HLM, noting that “all three political declarations should be viewed as complementary and interlinked.”

### GHS core functions

Core functions of GHS provide particular insights into how GHS is being operationalized in the PD as specific actions. These can be grouped in three ways: (1) outbreak preparedness, (2) health emergency response, and (3) a One Health approach.

## Outbreak preparedness

One category of GHS core functions described across PD texts centers on outbreak preparedness. PD1 highlights that “lessons and innovations from the COVID-19 pandemic are providing opportunities to scale up PHC approaches, for example by using digital health technologies, and promoting public health literacy, self-testing and use of community-based services.” This emphasis on essential public health functions linked to preparedness is reflected in subsequent drafts. PD2 advocates for “countering vaccine hesitancy […] to prevent outbreaks as well as the spread and re-emergence of communicable diseases,” “public health surveillance,” and ensuring that “essential public health functions are among the core components of preparedness for health emergencies.” PD3 introduces “risk communication and community engagement” as well as “prevention, early detection and control of diseases.” Additionally, by recommending “continuity of care in national and cross-border contexts,” PD3 visibly promotes a UHC approach in an area traditionally covered by GHS. PD4 builds on earlier calls to “implement the IHR (2005)” and “[integrate] disaster and health risk management systems.” Finally, the ADP largely retains these outbreak preparedness functions, and importantly inserting language on their affordability and accessibility as part of strengthening the “resilience of health systems.”

## Health emergency response

The PD also incorporates GHS core functions through language on health emergency response. PD1 notes that “inequitable access to medical products is among the main causes of financial hardship,” urging the provision of “critical countermeasure[s]” such as “COVID-19 vaccination [for] high priority groups,” “recovery and strengthening of the essential immunization programme,” and “essential services relating to HIV […] to end AIDS as a public health threat.” PD2 further calls for “integrated service delivery [for] HIV/AIDS, tuberculosis, malaria, hepatitis, and neglected tropical diseases,” while specifically advocating for “the production and timely and equitable distribution of COVID-19 vaccines, therapeutics, diagnostics and other health technologies.” Added language on “availability and equitable” access in PD3 concerning the “manufacturing, regulation, procurement,” and deployment of essential medical products and services is retained in PD4 and further strengthened in PD5, which “promote[s] the transfer of technology and know-how and encourage research, innovation and commitments to voluntary licensing” as critical components of pandemic response.

## One Health approach

While PD1 briefly mentions One Health as part of an “integrated health tool […] for national strategic health planning and costing,” subsequent PD drafts significantly develop a focus on this key aspect of GHS. For example, PD2 affirms the need to “enhance cooperation at the national, regional and global levels for an integrated and systems-based One Health approach.” PD2 goes on to detail specific features of One Health that are vital for achieving UHC, including “to improve the prevention, monitoring, detection, control and containment of zoonotic diseases and pathogens, threats to health and ecosystems, the emergence and spread of antimicrobial resistance, and future health emergencies, by fostering cooperation and a coordinated approach between the human health, animal health and plant health sectors, environmental and other relevant sectors.” Successive iterations in PD3, PD4, and PD5 largely retain the same language, and more broadly urge Member States “to adopt an all-hazard, multisectoral and coordinated approach to prevention, preparedness and response for health emergencies.”

## Discussion

This analysis advances current interpretations of GHS and UHC norms by examining how they are converging following the COVID-19 pandemic, tracing their expression and influence on two key negotiation processes – the WHO Pandemic Agreement and the 2023 UNGA Political Declaration on Universal Health Coverage. The findings provide three major insights: (1) the COVID-19 pandemic catalyzed a policy window uniquely favorable to accelerating normative convergence between GHS and UHC; (2) convergence between GHS and UHC norms was advanced through increased complementarity and interdependency between their respective discourse and core functions; and (3) sustaining GHS and UHC convergence remains a dynamic and contentious process heavily influenced by political and operational trade-offs.

This study highlights the hidden role of incremental and implicit shifts in shaping global health norms (rather than more visible advancements through explicit references). By detailing a nuanced ‘politics of integration,’ these findings offer practical lessons for policymakers and diplomats seeking synergistic approaches to strengthen GHS and UHC. It also provides fresh insights for foreign policy researchers studying norm theory in contested policy environments, who seek to understand the understudied, fluid process of normative convergence between two sets of influential norms and their associated political dynamics via diplomatic channels.

### COVID-19 as a catalyst for GHS and UHC convergence

This analysis suggests that the COVID-19 pandemic created a policy window particularly favorable for normative convergence between GHS and UHC. While key actors like WHO had already begun to connect these norms prior to the pandemic, increased international cooperation and political momentum driven by the crisis accelerated this phenomenon. The draft texts demonstrate that negotiators viewed COVID-19 as a driving force for both agreements; the PA acknowledged “serious shortcomings in preparedness at national and global levels,” [73] while the PD emphasized that COVID-19 “created new obstacles to [.] the 2030 Agenda for Sustainable Development.” [80] In effect, both agreements indicated intent among negotiators to move beyond the status quo, ushering a reconceptualization of global health norms and a fast-tracking of joint GHS-UHC frameworks already underway prior to the pandemic [82, 83]. This was further influenced by reassessments sparked by the midpoint of the SDGs and pressure to “promote alignment and synergies” [78] across the HLMs Thus, repeated commitments to ‘coherence’ do not appear incidental, but rather deliberate insertions intended to alleviate a politically-fraught normative landscape, with the PA and PD striving to advance simultaneous global health goals.

The emerging themes, particularly the promotion of equity and resilience frames, demonstrate new overlapping priorities following COVID-19 which favored convergence between GHS and UHC in ways previously not possible. For instance, the striking inequalities in accessing pandemic countermeasures appear to have enabled a repositioning of ‘equity’ as a core objective of the PA [71] – despite the reality that GHS documents historically privilege national security interests over human rights [84]. Thus, in a notable departure from previous GHS agreements (e.g., IHR), PA negotiators centered equity (a concept “hard-wired into the definition of universal health coverage” [76]) as a key discursive tool in response to challenges such as vaccine hoarding. This helped expand the scope of the PA beyond traditional GHS capacities, including acknowledging how pandemics affect vulnerable populations. The prominence of equity frames also facilitated an entry point for other UHC norms into the GHS regime. For example, although GHS is conventionally operationalized via state-centric international security frameworks, equity framings reconfigured PA priorities to more carefully consider other UHC core functions (e.g., affordability of medical products, SDH). Meanwhile, given that ‘resilience’ is primarily utilized in the context of health emergencies [16], its promotion in the UHC-focused PD texts carried important associations with GHS, such as a focus on existential threats and infectious disease narratives. Moreover, resilience frames were strategically employed to help bridge implementation between GHS norms at an international level (e.g., pandemic preparedness) and UHC norms at a community level (e.g., PHC approach).

This demonstrates two important lessons. First, in the foreign policy community, discussions of norm development often assume that fraught normative environments inevitably lead to further fragmentation and silo-isation [85]. Yet in the case of GHS and UHC, we see that such contested landscapes may actually *foster* normative convergence when there is scope for overlapping priorities to build consensus, or when the status quo appears insufficient and policy constraints push stakeholders to work in new, more collaborative ways. This reflects prevailing theories of risk society, which posit that perceptions of risk during crises help to encourage policymakers to achieve consensus [86]. As the world faces increasingly multifaceted challenges in an era of “polycrisis” [87] – from climate change to rising inequality to armed conflict – fostering normative convergence in this way between multiple health and foreign policy goals may provide a strategic path for health diplomats to collectively address interconnected, ‘wicked’ problems. Second, equity and resilience, given their transecting features, may serve as overarching normative frames for future global health efforts. Their co-promotion in both PA and PD negotiations (e.g., “taking into account the need for equity and resilience” [73]) opened the door for joint elaboration of GHS and UHC norms by serving as the foundation upon which their respective discourse and core functions could be meaningfully introduced and debated – *together*. Furthermore, the preservation of equity and resilience across successive drafts serves as a testament to the significant normative weight they carry both individually and jointly. Just as powerful normative frames like ‘inclusivity’ and ‘integration’ [39] emerged halfway through the MDGs to significantly influence subsequent global health policy, ‘equity’ and ‘resilience’ may be well-positioned as powerful normative frames for global health advocates to leverage in future initiatives.

### Towards a shared GHS-UHC normative framework

In the wake of COVID-19, there was indeed notable convergence between GHS and UHC norms, indicated by: (1) increased complementarity (diffusion of UHC norms within the PA and diffusion of GHS norms within PD), and (2) increased interdependency (interlinkages between GHS and UHC norms that demonstrate cross-sectoral awareness). The establishment of a shared normative framework linking the discourse and core functions of GHS and UHC in these major international agreements portends a significant development for global health diplomacy – laying the groundwork for the emergence of a new ‘hybrid norm’ between GHS and UHC.

Text segments which specifically situate GHS and UHC together serve as some of the clearest examples of norm convergence. For example, early drafts of the PA repeatedly urged States to develop “health systems for UHC and health security,” while early drafts of the PD advocated “a foundation for universal health coverage, health security.” [76] Although subsequent versions slowly phased out such explicit references to jointly advance GHS and UHC, their significance in the foundational drafts of both the PA and PD appears to indicate that States (at least initially) regarded them as interconnected priorities. This is notable because previous international agreements centered on GHS or UHC lacked a comparable level of integrative language at their outset, signifying a novel shift in the co-conceptualization of both norms [67]. 

The removal of many of these most obvious manifestations of GHS-UHC integration in subsequent drafts may be perceived by some as a failure to fully realize normative convergence. Indeed, the disappearance of key elements of both norms during negotiations provides evidence of a significant degree of contestation between how GHS and UHC might ultimately be expressed. However, *implicit* references to GHS and UHC convergence – through novel interlinkages between their underlying discourse and core functions – offer equally powerful insights into how global health norms evolve. We argue that rather than highly visible commitments which explicitly reference two norms together, the process of normative convergence may often involve more subtle advancements, affirming the oft-overlooked value of radical incrementalism [88] in progressing and reshaping global health policy, diplomacy, and governance.

Discourses used to implicitly promote UHC – equity, human rights, and SDH – exerted a profound influence on the scope of the GHS-focused PA. Equity considerations allowed for commitments aimed at mitigating disparities between high-income and low-income countries, rights-based narratives stressed inclusivity and UHC as “the practical expression of the right to health,” [7] and SDH approaches focused on “protect[ing] lives and livelihoods” [73] demonstrated a long overdue focus on the socioeconomic needs of communities during pandemics. Together, these themes represented a firm ideational commitment to UHC, even while explicit language on UHC diminished. Furthermore, core functions associated with UHC – prioritizing vulnerable populations, ensuring affordability and accessibility of health products, and strengthening PHC – facilitated the operationalization of UHC within a GHS framework. This is significant, as previous GHS texts seldom addressed topics like price transparency, routine service delivery, and equitable access to countermeasures. Even core capacities referenced from the IHR (2005) (a hallmark of GHS), such as zoonotic events and laboratory networks, often drew on UHC for their operationalization in PA drafts, such as embedding community engagement into One Health initiatives and recommending accessibility provisions for pandemic-related diagnostics. The integration of UHC core functions into the GHS regime represents a significant paradigm shift for global health policymakers, fundamentally reshaping the scope of GHS norms as necessitating a simultaneous advancement of (at least some) central UHC principles and obligations for States.

Meanwhile, discourses implicitly promoting GHS in the PD – resilience, existential threats, and infectious diseases – elevated UHC to the realm of ‘high politics’ purportedly occupied by GHS [89] in an effort to rejuvenate stalled progress. This often relied on a “grammar of securitization,” [90] utilizing language like “threat” and “shadow pandemic” even when referring to health conditions primarily associated with UHC (e.g., non-communicable diseases). Core functions associated with GHS – outbreak preparedness, health emergency response, and One Health – underscore the profound impact COVID-19 had on the conceptualization of UHC. A robust emphasis on integrating PHC with traditional IHR core capacities and newer PPR functions like mitigating outbreak disinformation comprise much of the operational backbone of the document. Meanwhile, references to major GHS actors (e.g., Pandemic Fund) and multiple references to ongoing epidemics demonstrate that infectious disease control was viewed as an integral aspect of sustainable UHC. Finally, the inclusion of entire sections on One Health, integrated public health surveillance, and healthcare during armed conflicts – noteworthy additions rarely seen in previous UHC agreements – indicate substantial areas for converging global health governance across human, animal, environmental, and humanitarian health in ways that fundamentally infuse GHS into UHC initiatives.

### Barriers to sustaining convergence

Despite noticeable progress towards integrating GHS and UHC norms, mixed uptake over successive PA and PD drafts suggests that sustaining convergence from principle to practice remains a dynamic and contentious process. Both sets of documents demonstrated increasing normative convergence until their respective Zero Drafts, but lost many crucial linkages in subsequent iterations (i.e., reduced references to complementary norms). Scholars have previously described how, as negotiations approach deadlines, a variety of linguistic and strategic compromises may be sought by negotiators to facilitate consensus [91]. Our findings demonstrate this use of specific negotiation tactics (caveats and qualifiers, ‘palatable’ proxies, and forum-shifting), which, although applied for broader political purposes, ultimately limited the extent of GHS-UHC norm convergence possible in both international agreements. Mitigating these tradeoffs will be crucial for policymakers and negotiators seeking normative convergence in future diplomatic efforts.

Firstly, the insertion of caveats and qualifiers as negotiations progressed resulted in increasingly ambiguous commitments. While many preambular sections demonstrated relatively greater evidence of integrated discourse in principle, operative paragraphs in both agreements were eventually peppered with caveats like “as appropriate,” “in accordance with,” and “within available means and resources” in lieu of previous iterations which more concretely strengthened obligations. This pattern, explained by observers [92] as concessions to facilitate consensus, was applied to a range of topics beyond just GHS or UHC; however, their insertion undercut notable advancements in GHS-UHC convergence featured in earlier drafts. Additionally, later PA texts qualified references to UHC as only pertaining to PPR contexts (previous versions promoted GHS and UHC as twin goals for broader contexts), while the PD featured qualifiers like “potential” and “striving to”; these narrowed the scope and strength of commitments. While such linguistic amendments may be inevitable outcomes in consensus-based negotiations, this phenomenon nonetheless exhibited how normative convergence can quickly be undermined if operational language is weakened – an issue future negotiators should play close attention to.

Secondly, the replacement of direct references to GHS and UHC with less contentious substitutes demonstrates another way convergence can be undermined, a process documented by scholars in other similar international negotiations [93]. As time passed, negotiators in both drafting cycles were forced to cherry-pick specific aspects of complementary norms to retain (e.g., PHC in the PA, or One Health in the PD), rather than maintain explicitly-joint references or comprehensively advance the shared normative framework (i.e., integrated governance and financing for GHS and UHC was quickly abandoned). For example, while direct references to UHC were largely negotiated out of the PA, discourse themes like equity (which has been characterized as a “measurable component of UHC” [24]) could be used as a more ‘palatable’ proxy for UHC, thus implicitly expressing some aspects of the norm while avoiding some of the political baggage carried by the term. Meanwhile, various commitments to PPR enabled later PD texts on UHC to continue evoking GHS norms without explicit mention, given that PPR has been characterized as a more agreeable substitute for GHS in places where ‘security’ illicits negative connotations [6]. This was compounded by negotiators strategically ‘trading-off’ [94] explicit references to GHS or UHC in favor of other priorities as a source of leverage, particularly if more acceptable alternatives could be used in their place. For example, initial PA texts prominently emphasized UHC through discourses around human rights, but later versions scaled back provisions around community engagement – essentially handicapping meaningful implementation of UHC. The strategic use of proxies suggests a complex reality – that negotiators were largely united on the initial vision of aligning GHS and UHC norms, but divided on the extent to which they should be integrated and operationalized. Future policymaking and diplomacy aimed at fostering normative convergence, such as between GHS and UHC, should be weary of ‘trading’ away core principles and functions in pursuit of consensus, which may ultimately render final obligations meaningless.

Finally, given a politically-fraught environment, forum-shifting [95] was routinely used to mitigate deadlock, resulting in weakened GHS and UHC norm convergence. Concrete commitments on difficult topics were postponed under the justification of “policy coherence” with other processes, such as parallel IHR amendments and two other simultaneous UNGA HLMs on health. The text edits suggest that negotiators believed other venues may potentially yield better results or could facilitate trade-offs for disputed topics – indirectly diminishing normative convergence between GHS and UHC in areas that were particularly contentious. For example, while previous iterations of the PD had numerous explicit references to GHS, the final versions prioritized themes such as resilience, while ostensibly leaving more direct normative expressions of GHS for the HLM on PPR [96]. This strategic shift reveals a nuanced politicking in global health diplomacy, where considerations of coherence and synergies across concurrent high-level negotiations play a pivotal role in shaping uptake, with only the most politically-feasible aspects of a norm ultimately retained. Furthermore, given that the preparatory documents of both sets of agreements (largely drafted by technical specialists at WHO) provided the most explicit language promoting GHS and UHC integration, advocates should consider how to preserve such negotiating texts *after* they leave the technocrats who drafted earlier iterations and enter the political realm of UN or country-level diplomats.

### Future implications

As Shany argues, the evolution of international norms and institutions is “ultimately deferential to State sovereignty and relative power considerations.” [97] Reflecting this realpolitik inherent in global health diplomacy, as negotiations progressed, disagreements around financing [98], health system capacity [99], and operationalization [100] chipped away at negotiating language that could have more meaningfully advanced an emerging GHS-UHC hybrid norm. Looking ahead, it will be important for global health diplomats and researchers to better account for the broader political and material factors which led to various tradeoffs that ultimately weakened GHS-UHC convergence. Furthermore, policymakers and advocates should more carefully consider the role of complex geopolitics and entrenched path dependencies when seeking greater synergies across previously-distinct agendas like GHS and UHC.

Amended language over successive drafts of both the PA and PD reveals how geopolitical rifts fuel major disagreements – many of which have implications on the convergence of GHS and UHC norms. For example, PA debates between high-income and low-income countries around contentious topics like PASB and CBDR fell along long-standing divides between related GHS and UHC norms [101], with the former pushing for strong language on human rights (UHC norm) and robust data-sharing during pandemics (GHS norm) while the latter urged clear obligations on richer nations to provide technologies and financial assistance to meaningfully ensure equity and resilience. The creation of the so-called Group of Equity [102] (conspicuously comprised of largely Global South countries) in the PA demonstrates this rise of regional or issue-based blocks trying to concretize specific aspects of UHC in an agreement that had originated among Global North countries. Weakened language and debates over operationalizing rights-based approaches suggest that diplomatic efforts were ultimately unsuccessful in moving from rhetoric to action – resulting in weakened GHS and UHC norm convergence. Similarly, geopolitics surrounding PD, particularly removal of language around climate change and armed conflict (which would have implicated greater convergence with the GHS norm regime), suggest that challenges remain in rectifying major rifts across the international community on legitimizing “security” norms in public health. As a result, only the most palatable and technocratic health interventions (i.e., non-controversial principles unencumbered by concrete obligations) were advanced during negotiations.

This also suggests a related consideration, with some scholars contending that entrenched path dependencies [103] may have made it challenging for negotiators to sustain proposed language outside of the primary regime they were operating under. The drastic reduction of direct references to UHC in the PA and GHS in the PD over time provide the most obvious manifestations of path dependencies reifying preexisting fragmentation, while trade-offs that reduced reciprocal discourse and core functions further demonstrate this phenomenon. However, Göpel suggests that radical incrementalism can provide an effective and sustainable path to break longstanding structural siloes which perpetuate path dependencies [104]. Global health diplomats and policymakers could therefore prioritize targeted policies, investments, and systems strengthening efforts that intentionally (even if incrementally) foster convergence between cross-cutting agendas like GHS and UHC.

## Conclusion

This paper tests the proposition that UHC may be shaping policy solutions traditionally negotiated in GHS spaces (and vice versa), by analysing the extent of convergence between both norms through two case studies: the WHO Pandemic Agreement and the 2023 UNGA Political Declaration on Universal Health Coverage. Using a multimethod qualitative analysis, we traced the promotion of UHC through three related discourse themes and three related core functions in the PA (and vice versa for expressions of GHS in the PD). Holistically analysing both discourse and core functions enabled a more nuanced view into the inclusion of GHS and UHC norms into previously-distinct spaces, and the complex path toward normative convergence. The findings demonstrate a transformative shift, with the post-COVID-19 context providing a policy window uniquely positioned to accelerate normative convergence between GHS and UHC. They also indicate that, while convergence between both norms was significantly promoted at the start of negotiations, sustaining a shared GHS-UHC normative framework was ultimately undermined by a variety of political and operational trade-offs, with ongoing debates and shifting language suggesting tenuous progress as a result of power politics. Health diplomats and policymakers should consciously reject such forms of constructed ambiguity which may weaken hard-won progress on normative convergence.

Mainstreaming UHC in the PA was novel given the agreement’s roots in the GHS regime. However, as attention increasingly turned to details of operationalizing equity, concrete commitments to UHC were gradually cut as a negotiating provision to achieve consensus on other more controversial articles. Meanwhile, GHS capacities were inextricably linked with the exercise of UHC in the PD, with PPR a central aspect. However, critiques about the efficacy of threat-based narratives which may override human rights protections require further deliberation. Moving forward, the narrowing of equity’s scope in the PA and the lack of consideration for “bad’ resilience [105] in the PD need careful consideration. Furthermore, evaluations are required to assess how effective the PA (which is still being negotiated) and PD ultimately are in terms of sustainably promoting a hybrid norm linking GHS and UHC. Additionally, the ability of normative convergence to disrupt chronic cycles of ‘panic and neglect’ well after a crisis has passed needs further study. Finally, as Payne argues, given the highly contested political contexts surrounding normative development, “it can be essentially impossible [.] for scholars in retrospect to ascertain the resonance of any particular frame or counterframe,” [51] portending future areas for research on the evolution of global health norms.

The research underscores the importance of incremental advancements in reshaping norms, and recognizes that in the absence of explicit commitments, expressions of what they *represent* may be just as important. In doing so, we highlight the significance of advancing key aspects of GHS and UHC norms through progressive realization of their underlying discourse and core functions, with specific principles or obligations persisting even where ideal wording or definitions may be lost. We also argue that in politically-fraught diplomatic negotiations, the process of norm convergence may not only be an inevitable outcome for achieving consensus, but can also offer strategic advantages by promoting synergies across previously-siloed domains. This constructive approach to global health diplomacy, which aims to simultaneously advance interconnected GHS and UHC priorities, requires negotiators to better articulate “a radical vision combined with an incremental approach” [106] by navigating complex geopolitics in global health (e.g., striking a balance between international solidarity on one hand and national self-interests on the other) while overcoming entrenched path dependencies (e.g., intentionally financing and operationalizing overlapping areas of GHS and UHC). Ultimately, we argue that states should strategically view norm convergence as being inherently within their interests (both in terms of bridging geopolitical divides and breaking siloed thinking) – and codify this through diplomatic mechanisms.

This novel study has traced normative convergence through two sets of international negotiations situated across two complementary norm regimes, offering important contributions to global health and foreign policy. Public health policymakers and advocates can pragmatically apply its lessons by synergistically advancing both GHS and UHC, including the active promotion of ‘equity’ and ‘resilience’ as overarching frames for future discourse and implementation. Given the tendency in global health to retreat into silos in the face of competing priorities, ensuring integrated goals and approaches between GHS and UHC will require diplomats to meaningfully incorporate these across the guiding principles, scope, and operative paragraphs of international health agreements – and to consider where the promotion of strategic convergence between GHS and UHC may be best suited given the political context. Meanwhile, our analysis advances theoretical insights on the dynamic process of normative convergence between two broader norm regimes, suggesting that the development of hybrid norms can be expected and indeed leveraged in other environments, particularly in contexts rife with politics and contestation (e.g., climate change, humanitarian crises).

There is a tendency among policymakers to assume that if we cannot see explicit references or concrete commitments, that progress is not happening. But the way norms evolve represents important precursors crucial to subsequent policy and practice; how we frame these concepts matters profoundly to the way we institutionalize and operationalize them. Global health scholars, practitioners, and diplomats must appreciate the incremental advancements in norms that more often characterizes progress in global health. This requires a “willingness to accept small changes that together accrete to create bigger change, one step at a time.” [106] In the context of GHS and UHC, this means pushing for progressive realization of their underlying discourse and core functions where the full codification of either norm appears untenable. As resources become constrained amidst an era increasingly characterized by polycrisis and hard security politics, this paper concludes that pursuing normative convergence as a way to address multifaceted challenges will be crucial to future global health diplomacy efforts – and potentially more productive and strategic in the long run.

## Data Availability

No datasets were generated or analysed during the current study.
